# Mogroside V Protects against Hepatic Steatosis in Mice on a High-Fat Diet and LO2 Cells Treated with Free Fatty Acids via AMPK Activation

**DOI:** 10.1155/2020/7826874

**Published:** 2020-04-30

**Authors:** Linghuan Li, Wanfang Zheng, Can Wang, Jiameng Qi, Hanbing Li

**Affiliations:** ^1^Institute of Pharmacology, Zhejiang University of Technology, Hangzhou 310014, China; ^2^Section of Endocrinology, School of Medicine, Yale University, New Haven 06520, USA

## Abstract

Previous studies presented various beneficial effects of mogrosides extract from *Siraitia grosvenorii*, which has been included in the list of Medicine Food Homology Species in China. Mogroside V (MV) is one of the main ingredients in mogrosides extract; however, whether and how MV improves impaired lipid metabolism in the liver remains to be elucidated. Herein, we investigated the therapeutic effects of mogroside V upon hepatic steatosis *in vivo* and *in vitro* and explored the underlying mechanisms. The results showed that MV significantly ameliorated hepatic steatosis in high-fat diet- (HFD-) fed mice. Furthermore, the increased protein expression of PPAR-*γ*, SREBP-1, and FASN and mRNA expression of *pparg*, *srebp1*, *scd1*, and *fasn* in the liver in HFD-fed mice, which contribute to *de novo* lipogenesis, were dose-dependently reversed by MV treatment. Meanwhile, MV counteracted the suppressed expression of PPAR-*α* and CPT-1A and mRNA expression of *atgl*, *hsl*, *ppara*, and *cpt1a*, thus increasing lipolysis and fatty acid oxidation. In addition, in free fatty acids- (FFAs-) incubated LO2 cells MV downregulated *de novo* lipogenesis and upregulated lipolysis and fatty acid oxidation, thereby attenuating lipid accumulation, which was significantly abrogated by treatment with Compound C, an inhibitor of AMP-activated protein kinase (AMPK). Taken together, these results suggested that MV exerted a pronounced effect upon improving hepatic steatosis through regulating the disequilibrium of lipid metabolism in the liver *via* an AMPK-dependent pathway, providing a potential lead compound candidate for preventing nonalcoholic fatty liver disease.

## 1. Introduction

Over the past several decades, infectious liver diseases have been restrained effectively by excellent policies and novel therapeutic approaches. While noninfectious chronic liver disease is still a huge public threat to human health, one of the most common forms of chronic liver disease is nonalcoholic fatty liver disease (NAFLD). NAFLD is referred to as a continuum of liver conditions, ranging from simple steatosis, nonalcoholic steatohepatitis (NASH), fibrosis to cirrhosis [1]. And obesity is one of the most important clinical factors strongly correlated with NAFLD due to overnutrition and lack of physical activity. In obese individuals, excessive plasmic nonesterified fatty acids are primarily delivered by dietary and adipose tissue lipolysis of triglycerides, a process that is regulated by insulin action and dysregulated under circumstances of insulin resistance, and thus pose a great challenge to the liver. When fatty acids are either overwhelmingly supplied or improperly disposed, they may lead to the accumulation of toxic lipid species that provoke further hepatocellular injury. In 2018, up to 25 percent of the world population is involved in NAFLD [2]. So far, the prevention and treatment of NAFLD have become a hot topic in the field of future therapeutic regimens, research, and development.

The hallmark of NAFLD, hepatic lipid accumulation, results from increased *de novo* lipogenesis in the liver and decreased fatty acid *β*-oxidation. FASN is a key enzyme in the *de novo* lipogenesis pathway that is responsible for the synthesis of excess fat in the liver of patients with NAFLD and regulated by SREBP-1 and PPAR-*γ*. CPT-1A is essential for fatty acid oxidation, a process that metabolizes fats and converts them into energy, which is regulated by a transcriptional factor PPAR-*α* [3]. The understanding of the pathophysiology of NAFLD/NASH has evoked the proposition of the multihit model including adipose tissue dysfunction causing the release of excess FFAs from the adipocytes, increased levels of inflammatory mediators promoting insulin resistance, lipotoxicity, oxidative stress, and endoplasmic reticulum stress, and depletion of the synthesis, and hepatic levels of polyunsaturated fatty acids (PUFA) as well [4, 5].

Mogrosides belong to cucurbitane-type glycosides, which are the major bioactive constituents derived from *Siraitia grosvenorii* fructus. The fruit of *Siraitia grosvenorii* has been traditionally used in the treatment of lung congestion, dry cough, sore throat, and constipation for thousands of years in China [6]. And mogrosides from *Siraitia grosvenorii* fruits possess several advantageous properties including low calories, low toxicity, and high sweetness, which are approximately 300 times sweeter than those of sucrose. Furthermore, emerging literature has elucidated that mogrosides display multiple pharmacological effects on cancer, oxidative stress, fibrosis, inflammation, allergic asthma, obesity, and diabetes [6–10]. Liu et al. have shown that mogroside-rich extract performs a pronounced lipid-lowering effect in plasma and liver from high-fat diet (HFD)/streptozotocin-induced diabetic mice [11]. Meanwhile, it has been reported that mogrosides can inhibit the progress of NAFLD in HFD-fed mice [12]. Mogroside V is one of the main components in the mogroside-rich extract from *Siraitia grosvenorii* fructus. However, whether mogroside V affects NAFLD has not yet been investigated, and the in-depth mechanism needs to be elucidated.

In this study, we investigated the effects of mogroside V upon hepatic steatosis in mice fed a high-fat diet (HFD) and LO2 cells treated with free fatty acids (FFAs) and further explored the molecular mechanisms contributing to the beneficial effects, providing experimental evidence for developing new drug candidates against NAFLD.

## 2. Materials and Methods

### 2.1. Chemicals and Reagents

Mogroside V (MV, HPLC > 98%, Figure S1) was obtained from Nanjing Plant Origin Biological Technology Co., Ltd. (Nanjing, China; CAS:88901-36-4). Dorsomorphin (Compound C) was supplied by MedChemExpress (purity: 99.65%, Jersey, USA; CAS: 866405-64-3). Palmitic acid, oleic acid, and 3-[4,5-dimethylthiazol-2-yl]-2,5-diphenyltetrazolium bromide (MTT) were provided by Sigma (St. Louis, MO, USA). Triglyceride (TG), nonesterified fatty acid (NEFA), total cholesterol (T-CHO), aspartate aminotransferase (AST), and alanine transaminase (ALT) assay kits were obtained from Jiancheng Bioengineering Institute (Nanjing, China). Insulin ELISA kit was from Shanghai Hengyuan Biological Technology Co., Ltd (Shanghai, China). AMPK and p-AMPK (phospho-Thr172) antibodies were purchased from Santa Cruz (Dallas, USA); PPAR-*γ*, PPAR-*α*, FASN, CPT-1A, and *α*-tubulin antibodies were supplied by Proteintech (Chicago, USA); SREBP-1 antibody was purchased from Novus Biologicals (Littleton, Colorado, USA). TRIzol reagent was purchased from Invitrogen (Carlsbad, USA); BeyoRT™ III cDNA Synthesis Kit and BeyoFast™ SYBR Green qPCR Mix were purchased from Beyotime Institute of Biotechnology (Jiangsu, China).

### 2.2. Animal Experimental Design

Male C57BL/6 mice were purchased from the Center of Experimental Animals of East China Normal University (Shanghai, China; license no. SCXK 2016-0004). All mice were acclimated to the laboratory for a week before the follow-up experiments and were randomly assigned to two groups: the control group (*n* = 9) was fed a standard chow diet (Zhejiang Academy of Medical Sciences, Hangzhou, China) for 18 weeks and received a daily oral gavage of sterile saline for the last 8 weeks, and HFD group (*n* = 36) was fed high-fat diet (Table S1 [13]) for 10 weeks and had more than 20% weight gain. And then the HFD group was randomly divided into four subgroups: HFD group, low-dose MV group, medium-dose MV group, and high-dose MV group; all groups continued to be fed high fat-diets with corresponding treatments for 8 weeks. The HFD group received a daily oral gavage of sterile saline; the low-dose MV, medium-dose MV, and high-dose MV group were orally administered with different dosages of MV (25, 50, and 100 mg/kg/day), respectively. Before sacrificed, all animals fasted and minim blood was collected from the tail vein to measure blood glucose concentration using Onetouch Ultraeasy (Johnson, New Jersey, USA). Blood was collected from the fundus venous plexus for biochemical analysis. All mice livers were instantly removed and weighed. Pieces of the livers were fixed in 4% paraformaldehyde solution for pathological analysis. And the remaining liver samples were collected, snap-frozen in liquid nitrogen, and stored at −80°C for further study. The procedures of animal experiments were approved by the Institutional Animal Care and Use Committee at Zhejiang University of Technology Laboratory Animal Center (SYXK (Zhe) 2017-0001) and were in compliance with the Guide for *the Care and Use of Laboratory Animal* published by the National Academy Press (Washington, DC, 1996).

### 2.3. Serum Biochemical Analysis

Blood samples were centrifuged at 4,000 g for 5 min to obtain serum. Serum NEFA, TG, T-CHO, AST, and ALT levels were determined by assay kits according to the manufacturer's instructions. The level of insulin was measured using an insulin ELISA kit following the manufacturer's protocol. The homeostatic model assessment of insulin resistance (HOMA-IR) was calculated using the following formula: fasting insulin concentration (mIU/ml) × fasting glucose level (mmol/L)/22.5.

### 2.4. Cell Culture and Treatment

The human LO2 (HL-7702) cell line was acquired from Procell Life Science & Technology Co., Ltd. (Wuhan, China). LO2 cells were cultured in RPMI-1640 medium supplemented with 10% (*v*/*v*) fetal bovine serum (Gibco, California, USA), 100 U/ml penicillin, and 0.1 mg/ml streptomycin in a humidified incubator under 5% CO_2_ at 37°C. The cells were seeded in 6-well cell plates, grown to around 75% confluence, and next incubated with serum-free medium for 12 h. Then, the cells were incubated with freshly prepared medium supplemented with FFAs-BSA solution for 24 h to induce hepatocyte steatosis. The medium is constituted by 0.33 mM oleic acid and 0.17 mM palmitic acid containing 1% fatty acid-free bovine serum albumin (BSA). And LO2 cells in different groups were given the corresponding treatments.

### 2.5. Cell Viability Assay

Cell viability was assessed using the MTT assay. The cells were seeded (4 × 10^3^ cells/well) in a 96-well cell culture plate, incubated for 8 h, and then treated with different concentrations of MV (15, 30, 60, and 120 *μ*M) for 24 h. The MTT was dissolved at 5 mg/mL in phosphate-buffered saline. After incubation, the medium was replaced with 200 *μ*L freshly medium and each well was added with 20 *μ*L of MTT solution. After 4 h, the solution was discarded, and dimethyl sulfoxide was added to dissolve the formazan crystal. Finally, the absorbance was measured at 490 nm on an ELISA plate reader (BioTek SynergyH1, Winooski, USA).

### 2.6. Histopathological Analysis

After fixation, liver specimens were embedded in paraffin and sliced into sections. Then, these sections were stained with hematoxylin-eosin (H&E) for histological examination. For Oil Red O staining, after fixation, the tissues were embedded in the optimum cutting temperature compound and stored at −80°C for 1 hour. Subsequently, frozen liver sections (6 *μ*m thick) were stained with 0.5% Oil Red O and stained with hematoxylin. Images were captured using light microscopy and analyzed using Image-Pro Plus 6.0. For Oil Red O staining of cell samples, the cells were washed once with phosphate buffer solution and fixed in 4% paraformaldehyde solution for more than 10 min. After fixation, cells were stained with 0.5% Oil Red O application fluid for 15 min at room temperature, washed, and stained with hematoxylin counterstaining for 3 min. In order to quantify the level of Oil Red O content, 60% isopropanol was added into each well and shaken at room temperature for 10 min. Finally, the absorbance was measured at 520 nm on ELISA plate reader.

### 2.7. Immunoblotting

Proteins of the liver or cells were extracted by radio immunoprecipitation assay lysis buffer containing protease inhibitor and phosphatase inhibitor. Protein samples were separated on 8% SDS-PAGE and transferred to PVDF membranes. Then, the membranes were blocked with 5% BSA solution for 2 h at room temperature. After blocking, the membranes were exposed to primary antibodies overnight at 4°C, washed with TBST, and incubated with appropriate secondary antibodies for 2 h at room temperature. Subsequently, the membranes were visualized by enhanced chemiluminescence method, and the intensity of the band was determined using ImageJ software.

### 2.8. Total RNA Extraction and Quantitative PCR

Total RNA was extracted from livers using TRIzol reagent. One microgram of total RNA was converted to cDNA using the BeyoRT™ III cDNA Synthesis Kit according to the manufacturer's instructions. The cDNA products were performed with SYBR Green qPCR Mix and corresponding primers (Sangong Biotech, Shanghai, China) on StepOne™ Real-Time PCR System (Applied Biosystems). Relative gene expression was normalized by the housekeeping endogenous gene *gapdh* and fold changes were calculated using the 2^−ΔΔCt^ method. The sequences of primers are listed in Table 1.

### 2.9. Statistical Analysis

All values are expressed as the mean ± standard error of the mean (SEM). One-way analysis of variance (ANOVA) with Tukey's post hoc test was adopted to evaluate the statistical significance of the difference between multiple groups, and *t*-test was used to assess the difference between two groups by GraphPad Prism 5. *p* < 0.05 was regarded as statistical significance.

## 3. Results

### 3.1. Effects of MV Administration on Physiological and Biochemical Parameters in HFD-Fed Mice

To investigate the therapeutic effects of MV on HFD-induced hepatic steatosis in mice, the changes of physiological and biochemical parameters were determined. As shown in Table 2, administration with high-dose MV significantly reduced the increase in body weight induced by HFD. A similar trend was observed in the change in liver weight. Meanwhile, high-dose MV treatment obviously decreased the levels of serum NEFA (*p* < 0.001), TG (*p* < 0.05), and T-CHO (*p* < 0.05) compared with HFD group, which were induced by prolonged feeding with HFD. Furthermore, the markedly elevated levels of serum glucose and insulin in mice fed with HFD were dose-dependently abrogated by MV treatment. Thus, HOMA-IR index, indicating the degree of insulin resistance, was also significantly lowered in the MV groups compared with the HFD group. In addition, MV could restore the serum levels of ALT and AST which are considered as important indicators of liver function and elevated in mice treated with HFD.

### 3.2. MV Ameliorated HFD-Induced Hepatic Steatosis in Histopathology

**Figure 1:**
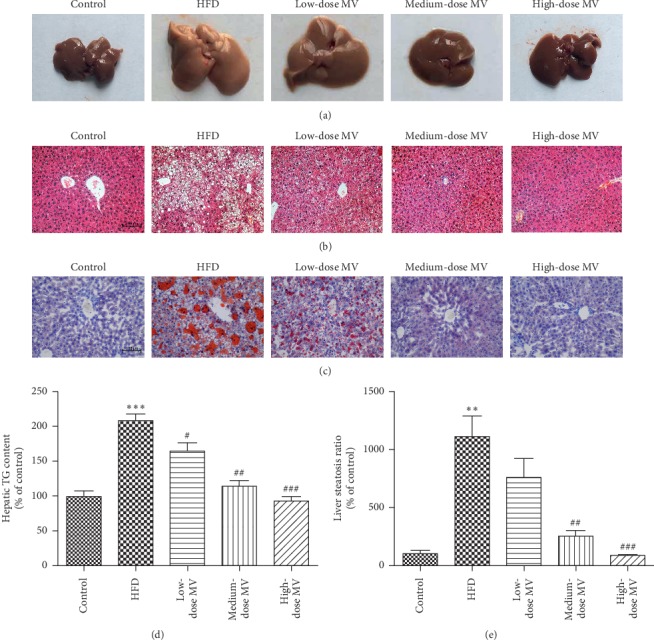
Effect of MV on hepatic histopathological changes in HFD-fed C57BL/6 mice. (a) Representative morphological images of the livers; representative photomicrographs of H&E-stained (b) and oil Red O-stained (c) liver samples; (d) quantitative analysis of hepatic TG content (*n* = 9); (e) quantification of oil Red O positive area (*n* = 4). Data are shown as mean ± SEM. ^*∗∗∗*^*p* < 0.001 vs. control; ^##^*p* < 0.01 vs. HFD; ^###^*p* < 0.001 vs. HFD.

To further investigate the effects of MV on fatty liver, we inspected the morphology and pathology of the liver. As exhibited in Figure 1, there were notable changes to the morphological feature of the liver; the liver color of the HFD group displayed pale yellow and that of groups supplemented with different dosages of MV changed from pale yellow to reddish brown. Concordant with the changes of morphology, H&E staining and Oil Red O staining showed that the lipid content of the livers was visibly reduced in the MV groups compared to the HFD-induced severe intrahepatic lipid accumulation from the aspect of the size and the number of lipid droplets. Furthermore, HFD-induced increase of intrahepatic TG accumulation was significantly inhibited by MV treatment. And quantitative analysis revealed that intrahepatic TG accumulation was dose-dependently reduced after the administration of MV suggesting the significant reduction of hepatic steatosis.

### 3.3. MV Regulated Lipogenesis, Lipolysis, and Fatty Acid Oxidation in the Liver of HFD-Induced Mice

**Figure 2:**
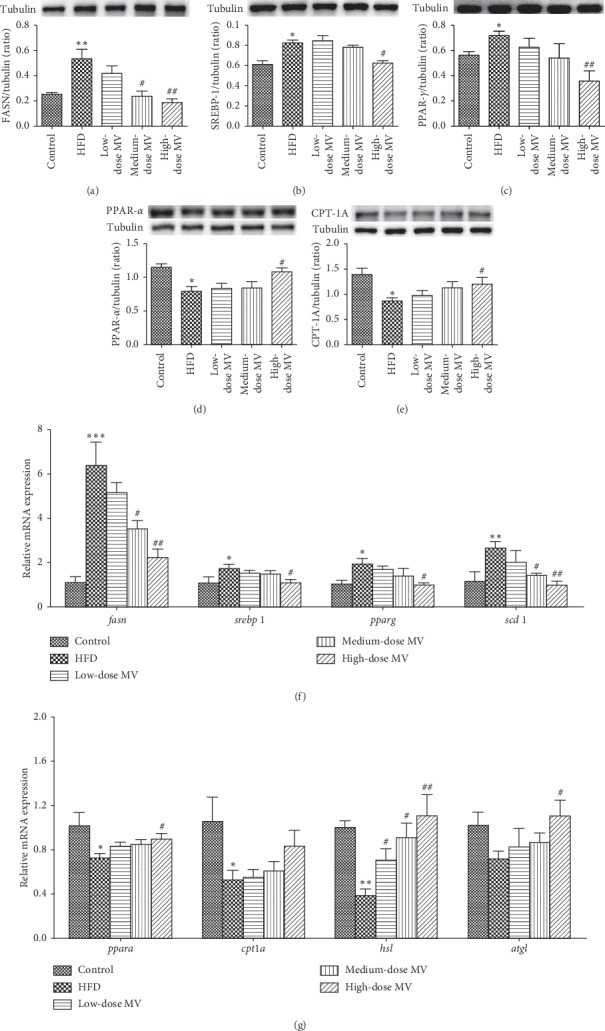
MV improved hepatic steatosis through inhibiting lipogenesis and promoting fatty acid oxidation and lipolysis in HFD-fed C57BL/6 mice. Representative immunoblot detection of FASN (a), SREBP-1 (b), PPAR-*γ* (c), PPAR-*α* (d), and CPT-1A (e) protein expression. (f) The mRNA expression of lipogenesis-relative genes including *fasn*, *srebp1*, *pparg*, and *scd1*. (g) The mRNA expressions of *ppara*, *cpt1a*, *hsl*, and *atgl* in the liver tissues of HFD-fed mice. Data are presented as mean ± SEM (*n* = 3-4). ^*∗*^*p* < 0.05 vs. control; ^*∗∗*^*p* < 0.01 vs. control; ^*∗∗∗*^*p* < 0.001 vs. control; ^#^*p* < 0.05 vs. HFD; ^##^*p* < 0.01 vs. HFD.

It is well known that lipid *de novo* synthesis, lipolysis, and fatty acid oxidation are essential events in the pathogenesis of hepatic steatosis. Therefore, we next measured the levels of protein and mRNA related to lipogenesis, lipolysis, and fatty acid oxidation in the liver. As illustrated in Figures 2(a)–2(c), the expressions of PPAR-*γ* and SREBP-1 and its downstream protein FASN were increased in NAFLD mice when compared with control group, whereas administration of high-dose MV significantly reduced the expression of these proteins.

Concordantly, MV contributed to a dose-dependent decrease of the gene expressions of *pparg*, *srebp1*, *scd1*, and *fasn* in contrast to those in NAFLD mice (Figure 2(f)). On the other hand, high-dose MV supplementation significantly enhanced the protein expression levels of PPAR-*α* (*p* < 0.05) and CPT-1A (*p* < 0.05) which were reduced by HFD. Similarly, quantitative PCR analysis presented that a reduction in mRNA expression of *ppara* and *cpt1a* in the liver tissues of HFD-fed mice was reversed by MV administration. In addition, we also evaluated the mRNA expression levels of lipolysis related genes including *atgl* and *hsl*. As shown in Figure 2(g), mice treated with MV exhibited a dose-dependent elevation in the hepatic expression of both *atgl* and *hsl* when compared with HFD group. These observations give a hint that MV treatment alleviates hepatic steatosis through the regulation of lipogenesis, lipolysis, and fatty acid oxidation in HFD-induced mice.

### 3.4. MV Alleviates FFAs-Induced Lipid Accumulation in LO2 Cells

**Figure 3:**
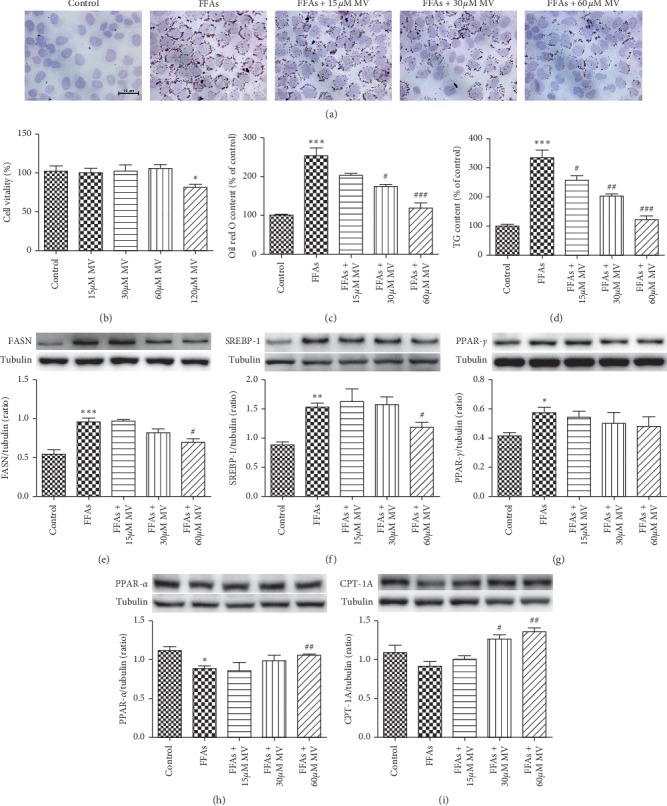
Effect of MV on FFAs-induced lipid accumulation in LO2 cells. (a) Representative photomicrographs of Oil Red O staining of LO2 cells; (b) cell viability (*n* = 6); (c) cellular Oil Red O content was extracted from cells and quantified (*n* = 6); (d) TG content was measured by the corresponding assay kit (*n* = 8); (e) FASN protein expression; (f) SREBP-1 protein expression; (g) PPAR-*γ* protein expression; (h) PPAR-*α* protein expression; (i) CPT-1A protein expression. All protein expressions were measured by western blotting assay (*n* = 3-4). Data are presented as mean ± SEM. ^*∗*^*p* < 0.05 vs. control; ^*∗∗*^*p* < 0.01 vs. control; ^*∗∗∗*^*p* < 0.001 vs. control; ^#^*p* < 0.05 vs. FFAs; ^##^*p* < 0.01 vs. FFAs; ^###^*p* < 0.001 vs. FFAs.

To further explore the effects and mechanisms of MV, LO2 cells were used to establish cell model of hepatic steatosis. As exhibited in Figure 3(b), incubation with 15, 30, and 60 *μ*M MV for 24 hours had no influences on cell viability, while 120 *μ*M MV significantly reduced cell viability. Then we examined the effects of MV upon lipid accumulation in FFAs-treated LO2 cells. As manifested qualitatively by Oil Red O staining and quantitatively by TG measurement in FFAs-treated LO2 cells, an obvious accumulation of intracellular lipid droplets was observed, which was dose-dependently alleviated by MV treatment (Figures 3(a), 3(c), and 3(d)).

To validate the potential mechanisms of MV on the modulation of lipid metabolism in FFAs-treated LO2 cells, the expression of key proteins to *de novo* lipogenesis and fatty acid oxidation were analyzed. Consistent with *in vivo* study, FFAs treatment significantly upregulated the protein expression levels of FASN, SREBP-1, and PPAR-*γ*, which were dose-dependently suppressed by treatment with different concentrations of MV (Figures 3(e)–3(g)). Meanwhile, FFAs incubation led to decreased protein expression levels of PPAR-*α* and CPT-1A, which were significantly enhanced by MV incubation. Taken together, these findings imply that MV treatment inhibits *de novo* lipogenesis and enhances fatty acid oxidation in LO2 cells, thus reducing hepatic steatosis in cell model.

### 3.5. MV Improved Hepatic Steatosis through AMPK Activation

**Figure 4:**
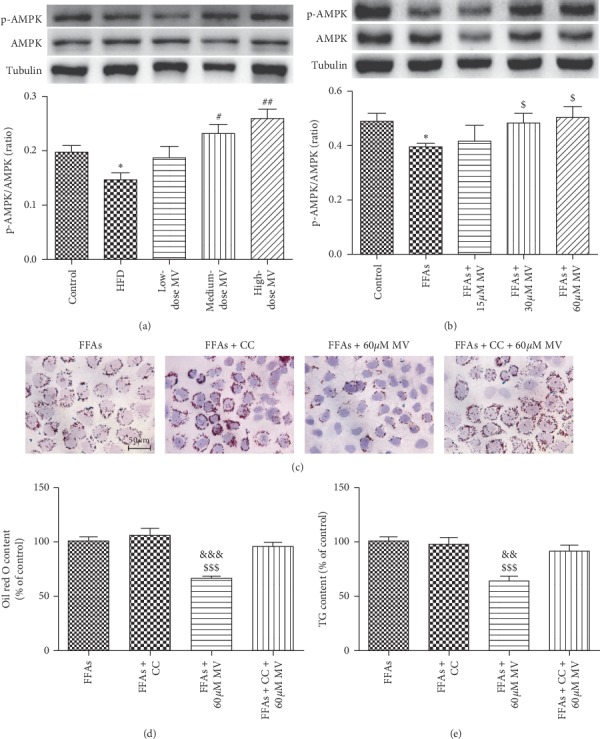
MV ameliorated lipid accumulation in liver tissues of HFD-fed mice and in FFAs-treated LO2 cells *via* AMPK. (a) Phospho-AMPK/AMPK protein expression in liver tissues (*n* = 4); (b) phospho-AMPK/AMPK protein expression in cells (*n* = 3); (c) Oil Red O staining of LO2 cells; (d) Oil Red O content quantification of LO2 cells (*n* = 6); (e) TG assay. All data are shown as mean ± SEM. CC: Compound c. ^*∗*^*p* < 0.05 vs. control; ^#^*p* < 0.05 vs. HFD; ^##^*p* < 0.01 vs. HFD; ^$^*p* < 0.05 vs. FFAs; ^$$$^*p* < 0.001 vs. FFAs; ^&&^*p* < 0.01 vs. FFAs + CC + 60 *μ*M MV; ^&&&^*p* < 0.001 vs. FFAs + CC + 60 *μ*M MV.

A considerable body of literature reports that AMP-activated protein kinase (AMPK) plays an important role in hepatocyte fatty acid metabolism including lipogenesis, lipolysis, and fatty acid oxidation [14, 15]. To determine the role of AMPK in MV treatment on hepatic steatosis, AMPK phosphorylation level was examined in liver tissues and LO2 cells. As demonstrated in Figure 4(a), compared with control group, AMPK phosphorylation level of HFD group was considerably decreased, which was dose-dependently upregulated by MV administration. Similar results were observed in LO2 cells incubated with different concentrations of MV (Figure 4(b)), implying that AMPK activation is involved in the therapeutic effects of MV on hepatic steatosis.

To verify whether MV ameliorates FFAs-induced steatosis *via* AMPK-dependent pathway, Compound C, an AMPK inhibitor, was adopted to treat LO2 cells. Intriguingly, Oil Red O staining exhibited that 60 *μ*M MV treatment significantly reduced the size and number of lipid droplets in FFAs-incubated cells, which was markedly abrogated by 5 *μ*M Compound C (Figure 4(c)). These results indicate that MV improved hepatic steatosis in an AMPK-dependent manner.

## 4. Discussion

Positive energy balance between excessive energy intake and less energy expenditure results in the increased incidence of overweight and obesity which is tightly associated with incidence of NAFLD. There is strong evidence that for each one unit increase in body mass index, the morbidity of NAFLD enhances by 13% to 38% and for every 1 cm increase in waistline, they increase by 3% to 10% [16]. The high levels of plasma FFAs are primarily originated from dietary fatty acids and adipose tissue lipolysis, which is vital to the pathological process of hepatic steatosis [17, 18]. Ectopic accumulation of hepatic lipids could attribute to boosted uptake of FFAs, strengthened *de novo* lipogenesis, decreased fatty acid oxidation, and suppressed very low-density lipoproteins export in hepatocytes [19], all alterations underpinning the occurrence of NAFLD. Therefore, the rationale for inhibition of *de novo* lipogenesis and enhancement of lipolysis and fatty acid oxidation could be therapeutic strategies for treating NAFLD. The mimic rodent model of hepatic steatosis can be induced by dietary including methionine and choline deficiency diet, HFD, cholesterol mingled cholate diet, and fructose diet. Among a series of the aforementioned rodent model, HFD-based animal model has been widely used for studies because of its similarity to human NAFLD in terms of the pathogenesis and alteration of metabolic parameters, especially the excess influx of FFAs into the liver causing lipotoxic process and promoting a hepatic proinflammatory state [20]. Therefore, in our study, hepatic steatosis was induced in HFD-treated mice to investigate the beneficial effects of MV upon hepatic steatosis including impaired *de novo* lipogenesis, lipolysis, and fatty acid oxidation.

The present study demonstrated that MV improved physiological, biochemical, and pathohistological changes, key characteristics of NAFLD, including weight gain, elevated NEFA, and hepatic steatosis, together with insulin resistance, which is coincident with the previous report that the major contributing factors to the pathogenesis of hepatic steatosis are insulin resistance and increased influx of free fatty acids in the liver [4]. In order to deeply understand the molecular mechanisms involved in the pathogenesis of NAFLD and therapeutic effect of MV, western blotting and quantitative PCR have been done to investigate the expression of key protein and genes related to lipogenesis, lipolysis, and fatty acid oxidation. Fatty acid synthase (FASN) and stearoyl-CoA desaturase-1 (SCD1) are lipogenic proteins required for *de novo* lipogenesis and encoded by the *fasn* and *scd1* gene, respectively, which are preferentially regulated by a transcriptional factor, SREBP-1. PPAR-*γ* is another transcriptional factor participating in the regulation of triacylglycerol synthesis in the liver, whereas PPAR-*α* regulates CPT-1A, which is responsible for the mitochondrial transport and oxidation of fatty acids. ATGL and HSL could contribute to the activity of liver lipolysis under physiological conditions. The results show that the increased expression of *pparg*, *srebp1*, *scd1*, and *fasn* and the suppressed expression of *atgl*, *hsl*, *ppara*, and *cpt1a* in the liver in HFD-fed mice were reversed by MV treatment. Results from FFAs-treated LO2 cells further indicating that MV ameliorated hepatic lipid accumulation through decreasing *de novo* lipogenesis, increasing lipolysis and fatty acid oxidation, which is consistent with previous knowledge that increased TG production and reduced fatty acid oxidation play important roles in NAFLD pathogenesis [21].

Furthermore, MV exerted a dose-dependent effect of increasing the AMPK phosphorylation, and the reduction of the size and number of lipid droplets in FFAs-incubated cells by MV was markedly abrogated by Compound C indicating that MV could improve hepatic lipid accumulation through activation of AMPK signaling pathway. AMPK acts as an important regulator of multiple metabolic pathways including hepatic lipid metabolism and its activation is considered as a potentially adversarial approach for the prevention and treatment of NAFLD [14, 22]. AMPK consists of *α*, *β*, and *γ* subunits. The threonine 172 is the major trigger point on AMPK*α*, which plays an important role in the process of AMPK activation [23]. Activation of AMPK regulates hepatic lipid metabolism through modulating several transcriptional factors, including SREBP-1, as well as PPAR-*α* and PPAR-*γ* [24, 25]. These transcriptional factors regulate genes responsible for *de novo* lipogenesis and fatty acid oxidation, which are two crucial events to adapt to fat accretion in the liver and to the development of NAFLD. In an individual with NAFLD, the level of *de novo* lipogenesis increased more than 3-fold compared with the normal individual [26].

## 5. Conclusions

**Figure 5:**
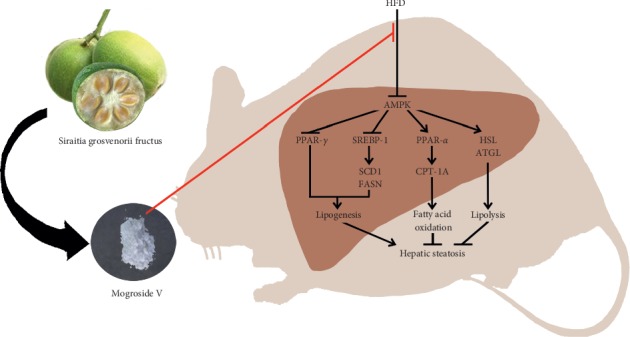
Summary of effects of mogroside V upon hepatic steatosis in mice fed an HFD.

In conclusion, MV ameliorates HFD-induced hepatic steatosis through AMPK activation which regulates SREBP-1, PPAR-*γ*, and PPAR-*α* and thus improves the imbalance between lipid acquisition and lipid removal (Figure 5), providing us with a potential lead compound for developing new drug. However, the deeper understanding of the mechanism of therapeutic effect of MV upon NAFLD and its druggability need to be elucidated.

## Figures and Tables

**Table 1:** Primer sequences for quantitative PCR.

Target genes	Forward primer (5′ ⟶ 3′)	Reverse primer (5′ ⟶ 3′)
*fasn*	CAGCAGAGTCTACAGCTACCT	ACCACCAGAGACCGTTATGC
*srebp1*	AAGCAAATCACTGAAGGACCTGG	AAAGACAAGGGGCTACTCTGGGAG
*pparg*	CGAGTGTGACGACAAGGTGA	CAGGCTGTTGGTCTCACAGG
*scd1*	TACACCTGCCTCTTCGGGATT	GCCGTGCCTTGTAAGTTCTG
*ppara*	AGGGTTGAGCTCAGTCAGGA	GGTCACCTACGAGTGGCATT
*cpt1a*	GACTATGTGTCCTGTGGCGG	CAAAGCGGTGTGAGTCTGTC
*atgl*	AGGACAGCTCCACCAACATC	TGGTTCAGTAGGCCATTCCT
*hsl*	TCAACTGGAGAGCGGATA	CTGAATAGGCACTGACACA
*gapdh*	ATGGTGAAGGTCGGTGTG	CATTCTCGGCCTTGACTG

**Table 2:** Physiological and biochemical parameters following 10-week administration of MV in HFD-fed C57BL/6 mice.

Parameter	Control	HFD	Low-dose MV	Medium-dose MV	High-dose MV
Body weight (g)	25.27 ± 0.40	40.82 ± 1.97^*∗∗∗*^	37.24 ± 1.82	36.40 ± 2.33	33.12 ± 1.97^#^
Liver weight (g/Kg b.w.)	41.69 ± 1.09	31.38 ± 0.76^*∗∗∗*^	31.40 ± 0.79	32.63 ± 0.52	34.88 ± 1.12^#^
NEFA (*μ*mol/L)	334.6 ± 13.8	500.2 ± 19.4^*∗∗∗*^	458.0 ± 21.6	408.9 ± 11.4^###^	409.0 ± 9.0^###^
TG (mmol/L)	0.79 ± 0.06	1.11 ± 0.07^*∗∗*^	1.09 ± 0.07	1.07 ± 0.03	0.93 ± 0.05^#^
T-CHO (mmol/L)	2.54 ± 0.20	5.76 ± 0.35^*∗∗∗*^	5.46 ± 0.22	5.50 ± 0.35	4.51 ± 0.32^#^
Blood glucose (mmol/L)	3.88 ± 0.26	6.86 ± 0.32^*∗∗∗*^	5.9 ± 0.29	5.09 ± 0.31^##^	4.11 ± 0.44^###^
Insulin (mIU/L)	3.91 ± 0.24	5.46 ± 0.21^*∗∗∗*^	4.88 ± 0.14^#^	4.72 ± 0.22^#^	4.27 ± 0.23^##^
HOMA-IR	0.66 ± 0.04	1.67 ± 0.14^*∗∗∗*^	1.29 ± 0.07^#^	1.06 ± 0.07^##^	0.78 ± 0.10^###^
AST (U/L)	15.87 ± 1.06	22.34 ± 2.79^*∗*^	23.25 ± 1.87	20.30 ± 2.73	18.76 ± 2.19
ALT (U/L)	20.41 ± 1.27	31.78 ± 3.42^*∗*^	25.67 ± 1.33	19.96 ± 2.98	18.68 ± 2.08^#^

Data are expressed as the mean ± SEM (*n* = 9). ^*∗*^*p* < 0.05 vs. control; ^*∗∗*^*p* < 0.01 vs. control; ^*∗∗∗*^*p* < 0.001 vs. control; ^#^*p* < 0.05 vs. HFD; ^##^*p* < 0.01 vs. HFD;^###^*p* < 0.001 vs. HFD.

## Data Availability

The data used to support the findings of this study are included within the article.

## Supplementary Materials

Supplementary Materials Figure S1: HPLC of mogroside V. Table S1: the ingredients of high-fat diet and standard chow diet.
