# Supplementary material for: Mogroside V Protects against Hepatic Steatosis in Mice on a High-Fat Diet and LO2 Cells Treated with Free Fatty Acids via AMPK Activation
Source: Evid Based Complement Alternat Med. 2020 Apr 30;2020:7826874. doi: 10.1155/2020/7826874 (PMC7210551; doi:10.1155/2020/7826874)
Supplement: Supplementary Materials — Figure S1: HPLC of mogroside V. Table S1: the ingredients of high-fat diet and standard chow diet. [file 7826874.f1.docx]

**Table S1** The ingredients of high-fat diet and standard chow diet

| Constituent | % (High-fat diet) | % (Chow diet) |
| --- | --- | --- |
| Protein | 11% | 11% |
| Fat (Crude fat, Lard) | 25% | 6% |
| Carbohydrate  (corn Starch, maltodextrin, sucrose and fiber) | 49% | 74% |
| Total | 91% | 91% |
| Ingredient | g/Kg (High-fat diet) | g/Kg(Chow diet) |
| Crude protein(≥80% protein) | 140 | 140 |
| Crude fat(85% fat) | 120 | 70 |
| Lard | 150 | - |
| Corn Starch | 150 | 300 |
| Maltodextrin | 200 | 400 |
| Sucrose | 150 | - |
| Fiber | 50 | 50 |
| Mineral mix (GB 14924.3-2010) | 30 | 30 |
| Vitamin mix (GB 14924.3-2010) | 10 | 10 |
| L-Cystine | 3.3 | 3.3 |
| Choline-bitartrate (44.4% Choline) | 2.8 | 2.8 |
| Total | 1006.1g | 1006.1g |


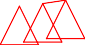


|  | 7.099  7.446  8.220 | | | | | | |
| --- | --- | --- | --- | --- | --- | --- | --- |
|  |  |  |  |  |  |  |  |
|  |  |  |  |  |  |  |  |
|  |  |  |  |  |  |  |  |
|  |  |  |  |  |  |  |  |
|  |  |  |  |  |  |  |  |
|  |  |  |  |  |  |  |  |

0.40

7.681

0.30

0.20

AU

0.10

0.00
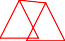


0.00 2.00 4.00 6.00 8.00 10.00 12.00

min

Analysis results

| Peak | Retention time (min) | Peak area (mAU*S) | Peak height (mAU) | % Peak area |
| --- | --- | --- | --- | --- |
| 1 | 7.099 | 17216 | 2337 | 0.74 |
| 2 | 7.446 | 11188 | 2961 | 0.48 |
| 3 | 7.681 | 2307277 | 446241 | 98.55 |
| 4 | 8.220 | 5513 | 1502 | 0.24 |

Figure S1. HPLC of mogroside Ⅴ. Peak 3 is mogroside Ⅴ.
